# Supplementary material for: Comparative Transcriptomics of Immune Checkpoint Inhibitor Myocarditis Identifies Guanylate Binding Protein 5 and 6 Dysregulation
Source: Cancers (Basel). 2021 May 20;13(10):2498. doi: 10.3390/cancers13102498 (PMC8161064; doi:10.3390/cancers13102498)
Supplement: Supplementary file 1 [file cancers-13-02498-s001.zip › cancers-1188625-supplementary.pdf]

cancers

MDPI

Supplementary Files

Comparative Transcriptomics of Immune Checkpoint Inhibitor Myocarditis Identifies Guanylate Binding Protein 5 and 6 Dysregulation

Daniel Finke, Markus B. Heckmann, Janek Salatzki, Johannes Riffel, Esther Herpel, Lucie M. Heinzerling, Benjamin Meder, Mirko Völkers, Oliver J. Müller, Norbert Frey, Hugo A. Katus, Florian Leuschner, Ziya Kaya and Lorenz H. Lehmann

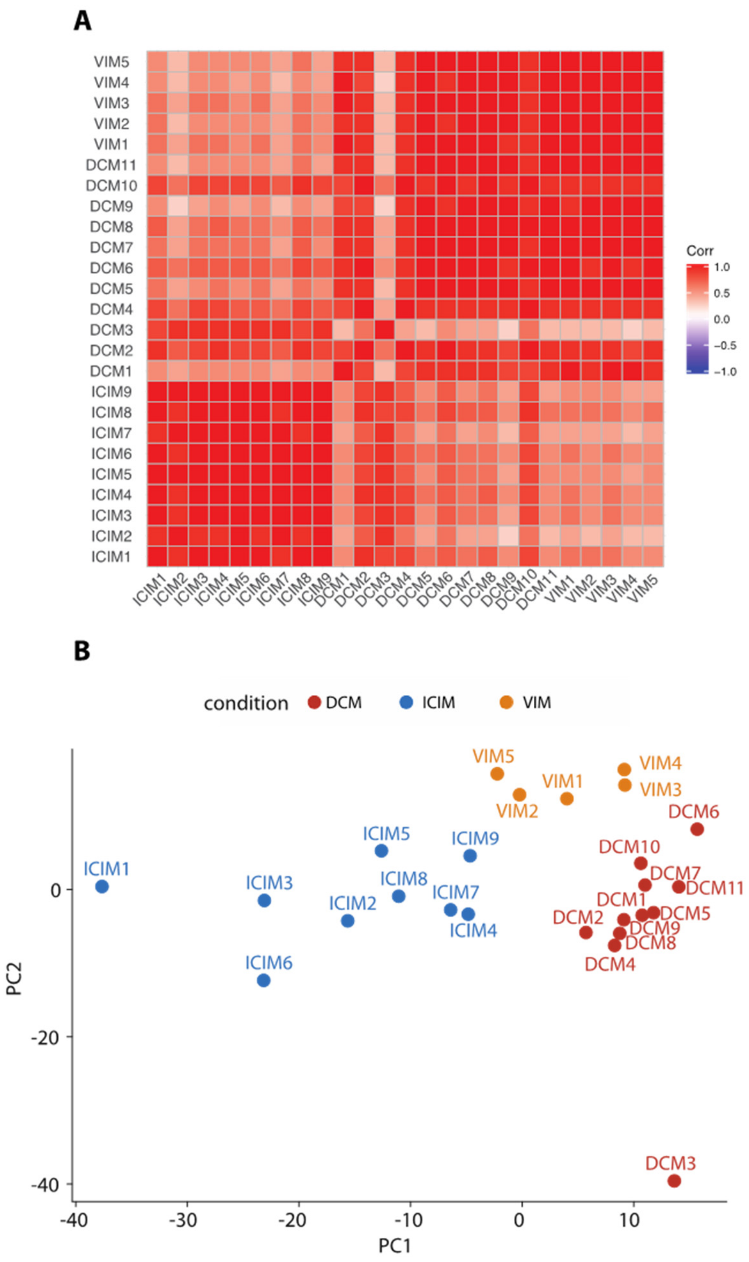

Figure S1. Clustering and Principal Component Analysis of ICIM, DCM and VIM. (**A**) Correlation plot and (**B**) principal component analysis (PCA) of the global read distribution of all analyzed RNAseq samples, ICIM: Immune checkpoint inhibitor associated myocarditis (n = 9), DCM: dilated cardiomyopathy (n = 11), VIM: virus-induced myocarditis (n = 5).

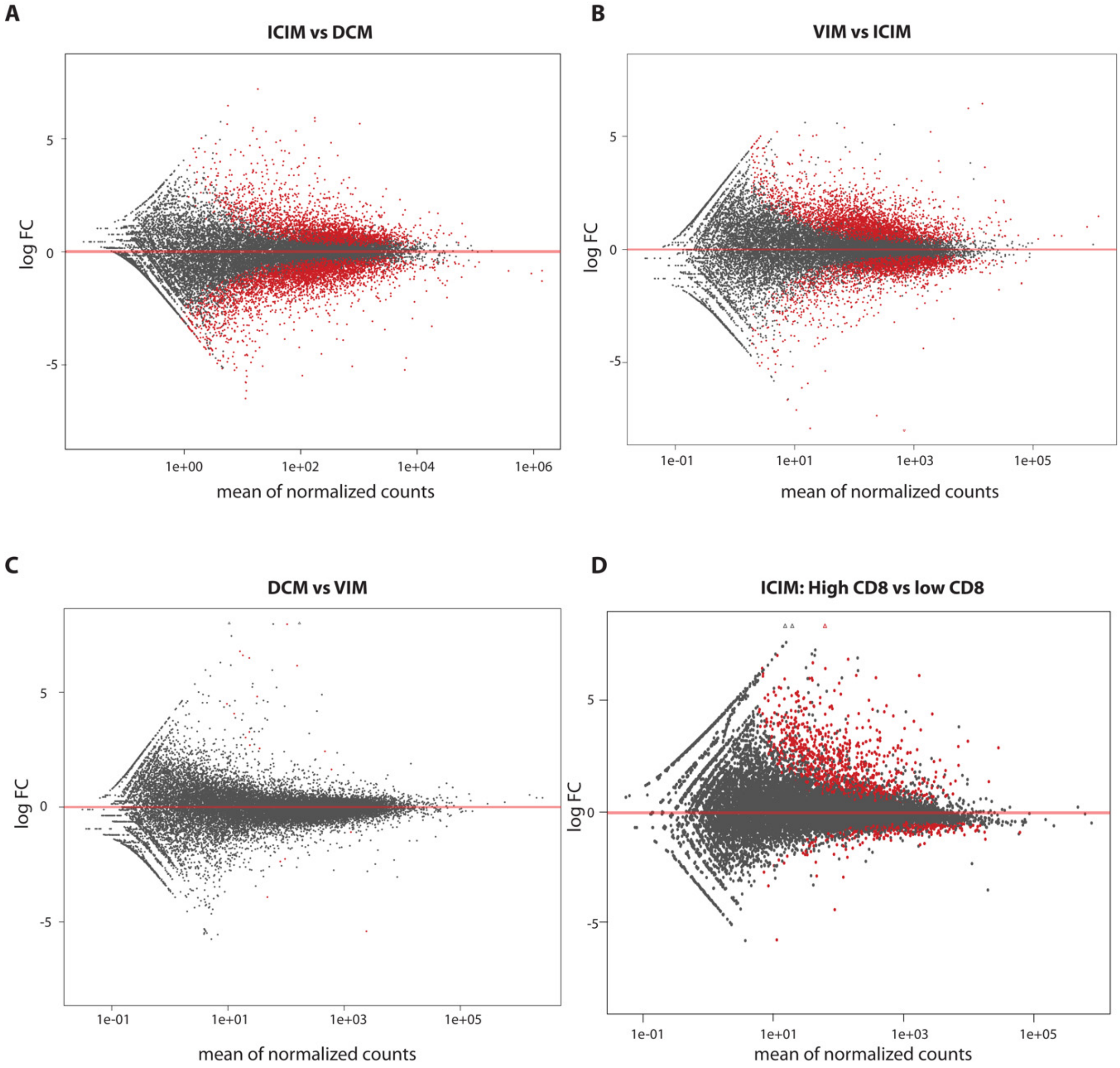

Figure S2. MA plots of the DESeq2 analysis. DESeq2 analysis (FDR < 0.05) of the comparisons (**A**) ICIM vs. DCM, (**B**) VIM vs. ICIM, (**C**) DCM vs. VIM and (**D**) high vs. low CD8a levels in ICIM, shown as MA plots (log2FC and mean of normalized counts as indicated).

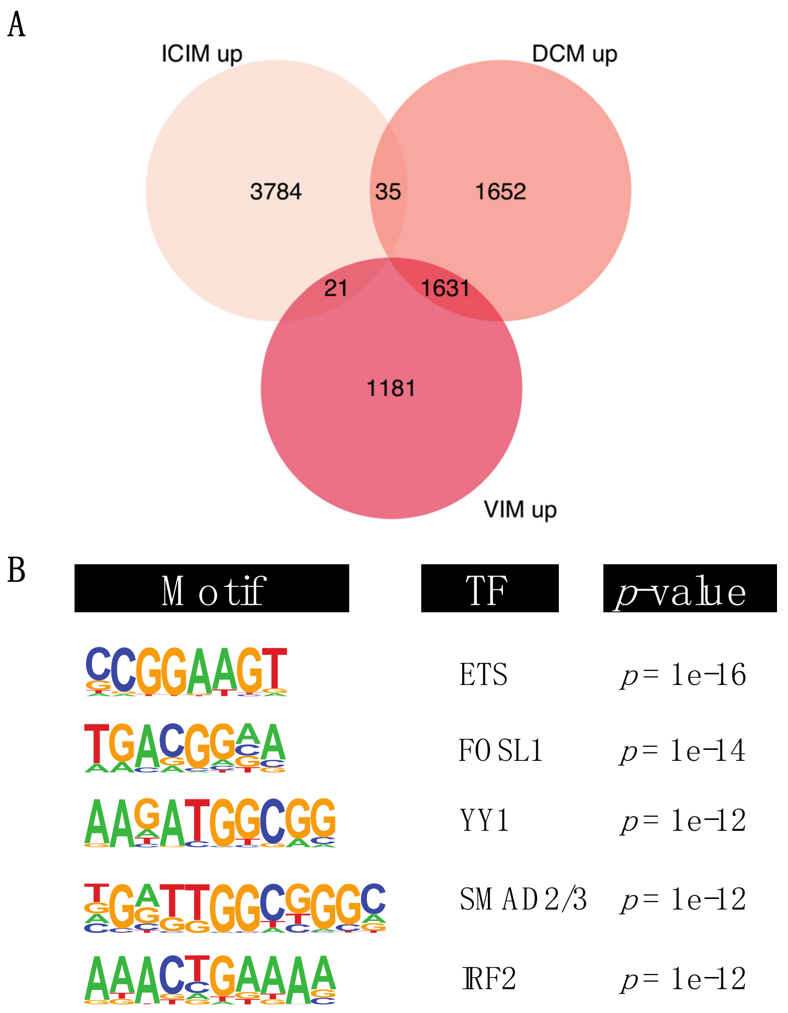

Figure S3. Overlap of upregulated genes in ICIM, DCM and VIM - TF analysis of ICIM-upregulated genes. (**A**) Venn diagram shows number of upregulated transcripts in ICIM compared to DCM and VIM, respectively.(**B**) De novo transcription factor (TF) binding analysis of the promoter region (-500bps to transcriptional start site) in ICIM upregulated genes. TF motif, name and *p*-value as indicated.

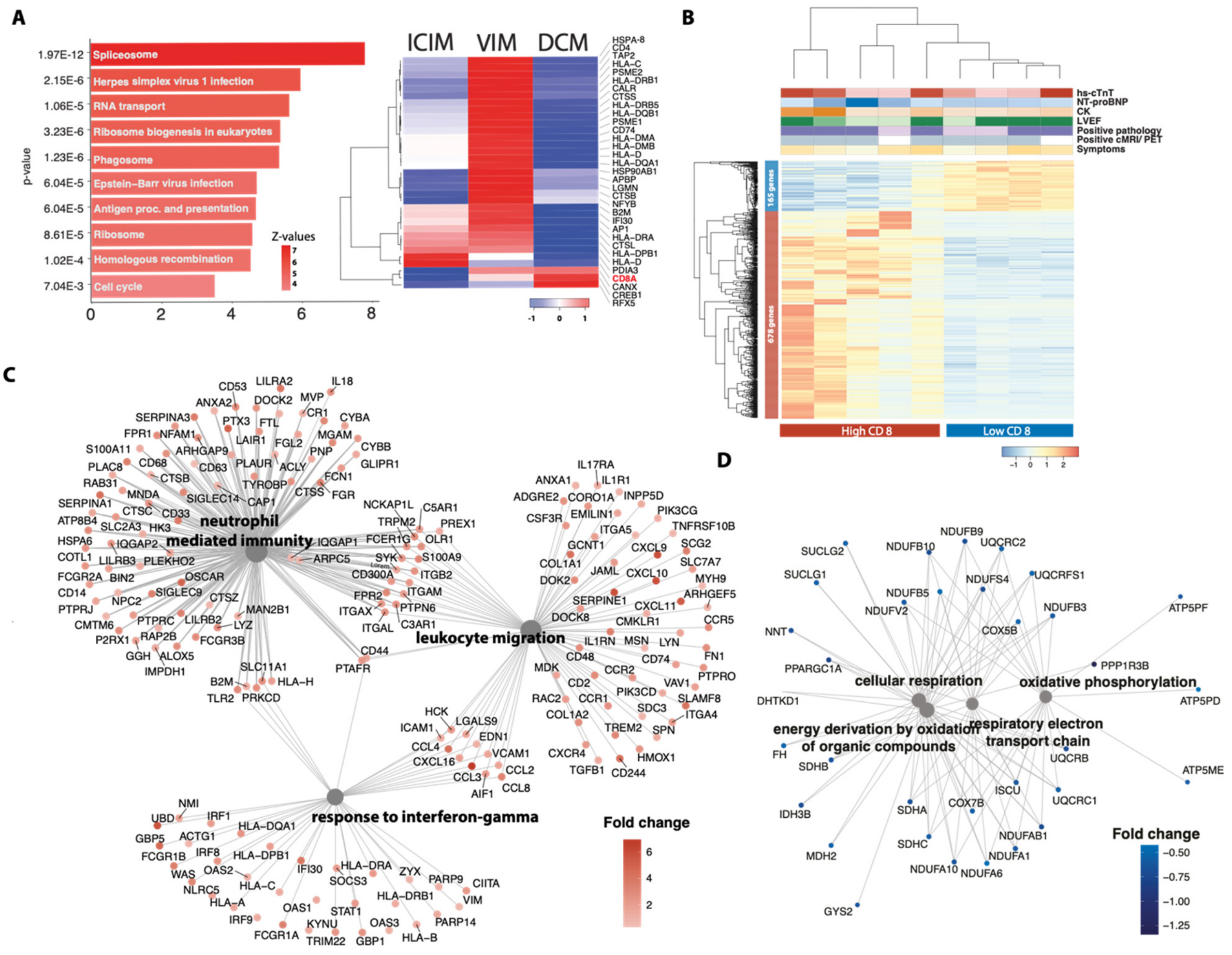

Figure S4. CD8-dependent gene program in ICIM. (**A**) KEGG pathway analysis of genes which are upregulated in ICIM, ordered according to their *z*-value. The heatmap shows genes of the cluster of antigen presentation (mean of each group). CD8 stands out as one of the most upregulated factors in antigen presentation after ICI treatment. (**B**) Heatmap clustered according to CD8 expression (median reads used as a cutoff) in the myocardial biopsies of ICIM patients. High initial troponin T (hs-cTnT), NT-proBNP and creatine kinase (CK) levels as well as preserved left ventricular ejection fraction (LVEF), positive pathology results, positive cMRI for myocarditis and existence of typical HF or ACS symptoms (according to NYHA and CCS classifications) are shown as indicated. Color code indicates *z*-score. (**C**) Network of enriched genes in the high CD8 group. The three major Gene Ontology (GO) pathways (neutrophil mediated immunity, p = 2.32E−34, response to interferon gamma, p = 1,45E−26, and leukocyte migration, p = 5.20E−28) and their corresponding genes are shown. Fold change (log2) of the genes between high and low CD8 as indicated. (**D**) Interaction Networks of less enriched transcripts in CD8+ biopsies. The major Gene Ontology (GO) terms: cellular respiration, energy derivation by oxidation of organic compounds, respiratory electron chain transport and oxidative phosphorylation are shown with their corresponding genes. Log2 fold change of the genes as specified.

Table S1. Characteristics of patients who received ICIs without evidence of myocarditis. Hs-cTnT, NT-proBNP and CK are specified as median values with interquartile range, the initial and maximum values are shown. BMI: body mass index, CK: creatine kinase, cMRI: cardiac magnetic resonance imaging, CRP: C-reactive protein, Hb: hemoglobin, HCC: hepatocellular carcinoma, Hs-cTnT: high sensitivity cardiac troponin T, NSCLC: non-small-cell lung cancer, NT-proBNP: N-terminal B-natriuretic propeptide, SCC: squamous-cell carcinoma, LV: left ventricle, LVEF: left ventricular ejection fraction, RV: right ventricle.

Characteristic	ICIM negative
n	4
Age (median, IQR)	70.5 (68.75, 74.25)
Gender (male)	3 (75%)
BMI (median, IQR)	29.1 (27.4, 32.1)
Arterial hypertension	3 (75%)
Diabetes	0 (0%)
Hb (median, IQR)	12.7 (10.98, 14.25)
Creatinine (median, IQR)	0.77 (0.73, 0.91)
CRP (median, IQR)	54.85 (23.23, 82.1)
Melanoma	2 (50%)
NSCLC	1 (25%)
HCC	0 (0%)
Kidney cell carcinoma	0 (0%)
SCC	0 (0%)
Thymoma	0 (0%)
Uterus carcinoma	0 (0%)
Pleural mesothelioma	1 (25%)
Nivolumab	0 (0%)
Nivolumab/ Ipilimumab	0 (0%)
Pembrolizumab	4 (100%)
Cemiplimab	0 (0%)
Durvalumab	0 (0%)
Atezolizumab	0 (0%)
LVEF > 50%	2 (50%)
LVEF 40–e50%	1 (25%)
LVEF < 40%	1 (25%)
Abnormal ECG	2 (50%)
Initital hs-cTnT [ng/L] (median, IQR)	75 (39,118)
Initial NT-proBNP [ng/L] (median, IQR)	NA
Initial CK [U/L] (median, IQR)	186.5 (85.75, 311)
Max hs-cTnT [ng/L] (median, IQR)	92 (64.75, 187.25)
Max NT-proBNP [ng/L] (median, IQR)	NA
Max CK [U/L] (median, IQR)	200 (106, 478)
Positive biopsy result	0 (0%)
LV-biopsies	4 (100%)
RV-biopsies	0 (0%)
Positive cMRI/PET-CT	0 (0%)
Definite ICIM diagnosis (1)	0 (0%)
Probable ICIM diagnosis (1)	0 (0%)

(1) According to the criteria of ICIM published by Bonaca et al .

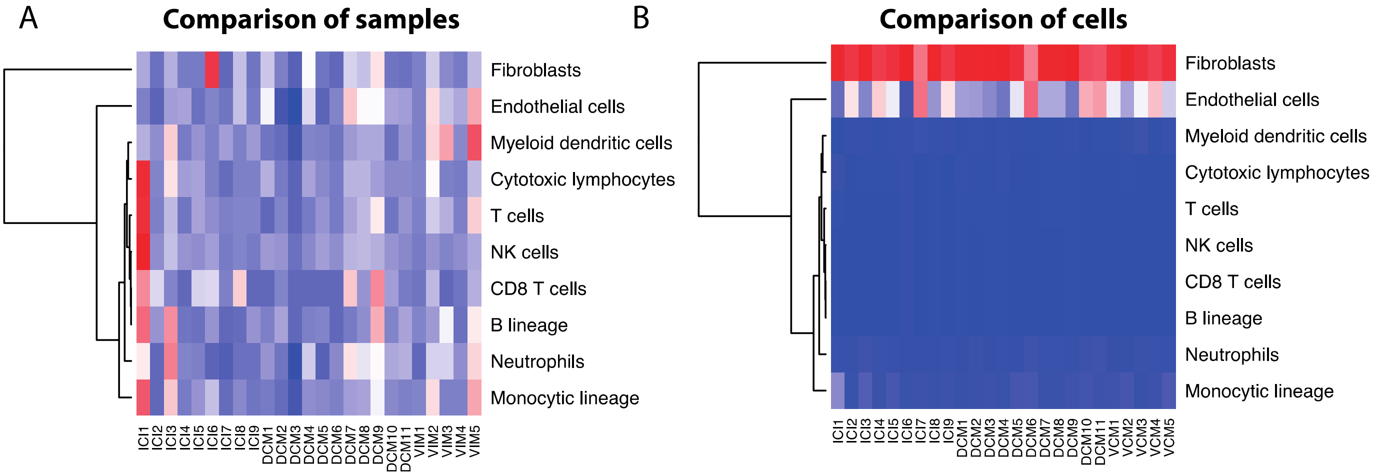

Figure S5. Bioinformatical estimation of immune and stromal cell infiltration (**A**) Enrichment of infiltrating cells and stromal cells was compared between the samples (ICIM, DCM and VIM) by a bioinformatical approach. (**B**) Relative comparison of the abundance of infiltrating immune cells and stromal cells showing predominance of fibroblasts and endothelial cells.

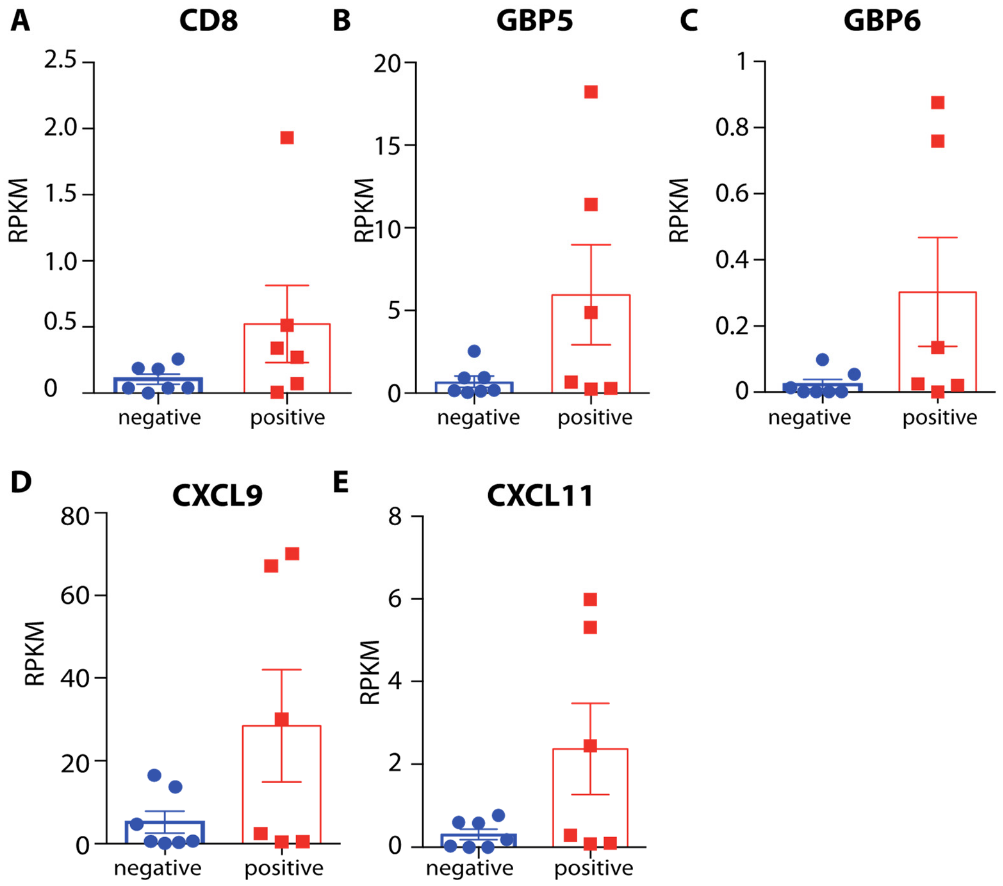

Figure S6. RNA expression levels in immunostaining negative and positive biopsies. The myocardial biopsies were divided into positive (detection of >14 lymphocytes/mm^2) and negative (no detection or < 14 lymphocytes/mm^2) results. The expression of (**A**) CD8, (**B**) GBP5, (**C**) GBP6, (**D**) CXCL9 and (**E**) CXCL 11 is shown for biopsies with negative and positive immunostaining results (RPKM: Reads per Kilobase per Million).
